# Tertiary lymphoid structures are associated with favorable survival outcomes in patients with endometrial cancer

**DOI:** 10.1007/s00262-021-03093-1

**Published:** 2021-10-23

**Authors:** Meng Qin, Junzo Hamanishi, Masayo Ukita, Koji Yamanoi, Shiro Takamatsu, Kaoru Abiko, Ryusuke Murakami, Taito Miyamoto, Haruka Suzuki, Akihiko Ueda, Yuko Hosoe, Akihito Horie, Ken Yamaguchi, Masaki Mandai

**Affiliations:** 1 Department of Gynecology and Obstetrics, Kyoto University Graduate School of Medicine, 54 Kawaharacho, Shogoin, Sakyo-ku, Kyoto, 606-8507 Japan; 2 Department of Obstetrics and Gynecology, Peking Union Medical College Hospital, Chinese Academy of Medical Sciences and Peking Union Medical College, Beijing, China; 3 Department of Obstetrics and Gynecology, National Hospital Organization Kyoto Medical Center, Kyoto, Japan; 4 Department of Gynecology, Shiga General Hospital, Shiga, Japan

**Keywords:** Tertiary lymphoid structures, B cells, Endometrial cancer, Survival outcomes, Immunohistochemistry, Immune response

## Abstract

Immunotherapy has experienced remarkable growth recently. Tertiary lymphoid structures (TLSs) and B cells may play a key role in the immune response and have a survival benefit in some solid tumors, but there have been no reports about their role in endometrial cancer (EC). We investigated the clinicopathological and pathobiological characteristics of the tumor microenvironment (TME) in EC. Patients with EC at Kyoto University Hospital during 2006–2011 were retrospectively included. In 104 patients with EC who met study inclusion criteria, 81 (77.9%) had TLSs, which consisted of areas rich in CD20^+^ B cells, CD8^+^ T cells, CD4^+^ T cells, and CD38^+^ plasma cells. The absence of TLS was independently associated with tumor progression (HR, 0.154; 95% CI, 0.044–0.536; *P* = 0.003). Patients with TLSs that included CD23^+^ germinal centers had better PFS. All tumor infiltrating lymphocytes were counted in the intratumor site. The number of CD20^+^ B cells was significantly larger in patients with TLSs than in those without TLS (*P* < 0.001). CD20^+^ B cells numbers were positively correlated with other TLSs. The larger number of CD20^+^ B cell was associated with better PFS (*P* = 0.015). TLSs and B cell infiltration into tumors are associated with favorable survival outcomes in patients with EC. They may represent an active immune reaction of the TME in endometrial cancer.

**Supplementary Information:**

The online version contains supplementary material available at 10.1007/s00262-021-03093-1.

## Introduction

Endometrial cancer (EC) is one of the most common gynecologic malignancies in the world [1]. The most important risk factors for EC are all related to an unbalanced increase in circulating estrogen, such as irregular menstruation, obesity, and exposure to tamoxifen [2]. Atypical endometrial hyperplasia (AEH) prior to EC is a continuously changing disease process. AEH is a precancerous lesion of EC, so most patients with EC can be diagnosed at an early stage [3]. However, the prognosis of patients with advanced EC and poorly differentiated histology is relatively poor, which makes therapy challenging [4]. Primary surgery followed by chemotherapy or radiotherapy has been the long-standing standard treatment for patients with EC, with or without hormonotherapy.

Recently, immunotherapy and targeted therapy have become alternative adjuvant treatment options as genetic evaluation in EC has become more widespread [5, 6]. Immunotherapy has experienced a remarkable growth in the last few years, including the use of immune checkpoint inhibitors and research on adoptive cellular transfer [5]. With the use of anti-programmed death 1 (PD-1) and anti-programmed death ligand 1 (PD-L1) agents, significant breakthroughs have been made in targeted immunotherapy involving T cells [7, 8]. Tumor cells interacting with immune cells in the tumor microenvironment (TME) mediate tumor formation, local invasion, and metastasis [9]. Tertiary lymphoid structures (TLSs), also called ectopic lymphoid tissues are B cell-rich structures in tumors that have recently been in the spotlight [10, 11]. There are many reports that TLSs exist in chronic inflammatory conditions, including autoimmune diseases, chronic infections, chronic graft rejection, and a variety of solid tumors [12]. TLSs serve as an effective site for tumor reactions in the human immune system and trigger an inflammatory response via infiltrating immune cells [13]. Recent studies have shown that proinflammatory cytokines and corticosteroid intake in the TME lead to TLSs [14]. The B cell pathway plays a key role in TLS formation and function [15]. The presence of TLSs has been demonstrated to be associated with beneficial survival outcomes in malignancies such as breast cancer [16, 17], lung cancer [18, 19], and colorectal cancer [20, 21]. However, there are no detailed reports about the relationships among TLS formation, local immune status, and clinical outcomes in patients with EC.

Therefore, we aim to investigate the clinicopathological and pathobiological characteristics of the TME in EC, in order to explore whether TLSs are present in EC and affect survival outcomes. We used immunohistochemistry (IHC) to explore the relationship between TLSs and tumor infiltrating lymphocytes (TILs), especially B cells.

## Materials and methods

### Patients and samples

This retrospective study included patients with EC who underwent primary treatment (total hysterectomy and bilateral salpingo-oophorectomy with or without pelvic lymphadenectomy and para-aortic lymph node dissection) at Kyoto University Hospital during 2006–2011. Patients with any of the following characteristics were excluded: (1) incomplete surgery and residual tumor; (2) neoadjuvant chemotherapy or neoadjuvant radiotherapy; and (3) rare pathological types other than endometrioid adenocarcinoma, uterine serous carcinoma (USC), or mixed type (endometrioid and serous adenocarcinoma). All patients provided written informed consent. This study was approved by the ethics committee of Kyoto University Hospital.

The following data were extracted from electronic medical records from the Hospital Information King System: patient information, clinicopathological characteristics, adjuvant treatment, risk factors, and survival outcomes. Risk factors included tumor size, depth of myometrial invasion, lymphovascular space invasion (LVSI), parametrial involvement, lymph node metastasis, and microcystic, elongated, and fragmented (MELF) pattern. Progression-free survival (PFS) and overall survival (OS) were the most important results for this study. PFS was defined as the time interval between the date of the first diagnosis and the date of disease progression. OS was defined as the time interval between the date of the first diagnosis and the date of death [22]. In terms of histology, low-grade disease consisted of G1 or G2 endometrioid adenocarcinoma, and high-grade disease consisted of G3 endometrioid adenocarcinoma, USC, or mixed type.

We included 10 randomly selected patients with normal endometrium (including 5 cases in the proliferative stage and 5 cases in the secretory phase) and 10 patents with AEH as control groups in this study.

### Immunohistochemistry

IHC was performed to detect CD8, CD20, CD4, CD38, and CD23 expression in patient samples using a standard protocol. The IHC conditions for each molecule in this study are shown in Table S1. Paraffin-embedded tumor blocks were cut into 4-μm-thick sections and then heated in a tissue-drying oven for 60 min at 60 °C. The tissue sections were deparaffinized in xylene in three steps of 15 min, 10 min, and 10 min, respectively. Next, the tissue sections were dehydrated with 99% (vol/vol) alcohol for 5 min, followed by 99% (vol/vol) ethanol for 2 min, 99% (vol/vol) ethanol for 2 min, and 70% (vol/vol) ethanol for 2 min and then rinsed with water. The antigen retrieval buffer consisted of 10 mM citrate buffer (pH 6.0) for all molecules. All sections were treated with methanol containing 0.3% (vol/vol) H_2_O_2_ for 15 min to block endogenous peroxidase activity, except for CD4, where methanol containing 3% (vol/vol) H_2_O_2_ was used. The sections were treated with normal mouse or rabbit serum (Histofine SAB-Po kit, #424,022 or 424,032, NICHIREI Biosciences Inc.) to block nonspecific binding of IgG. The sections were further incubated with corresponding primary antibody overnight at 4 °C. Next, the sections were stained with corresponding biotinylated secondary antibodies (NICHIREI Biosciences Inc.) for 30 min, followed by incubation with a streptavidin-peroxidase solution for 30 min. DAB (Sigma #D4418) was used to visualize peroxidase activity. Hematoxylin was used as a counterstain. Human tonsil tissue samples were used as positive controls for each molecule. Finally, the sections were dehydrated with alcohols in various percentages and xylene before coverslips were applied. All sections were washed in phosphate buffered saline (PBS), except for CD23, where PBS with 0.3% (vol/vol) Triton X-100 was used.

### Evaluation of TLS and immunohistochemistry

A gynecological pathologist and two gynecological oncologists who have abundant experience in pathology independently examined the slides with hematoxylin–eosin (HE) staining and IHC without any clinical information about the patient. A third reviewer was involved in a discussion to resolve differences. The presence, location, formation, and maturation of TLSs were assessed on each slide for all patients based on HE staining and IHC expression under the microscope. A TLS was defined as the area of ectopic lymphocyte aggregation larger than a microscopic field at 400x (0.03125 mm^2^). The TLS number was defined as the amount of TLS at 400 × microscopic field in one slide.

Tumor infiltrating cells were divided into infiltrating lymphocytes in a tumor site and infiltrating lymphocytes in stroma within a tumor area according to their location. In this study, all TILs were counted in the intratumor site. We did not count immune cells in TLSs as TILs. CD8^+^ T cells, CD20^+^ B cells, and CD4^+^T cells can be evaluated by the positive cell count method based on membrane proteins. For example, the evaluation steps for CD8^+^ T cells were: (1) distinguishing the intratumor area; (2) selecting and counting 10 fields with a large number of CD8^+^ T cells with a 400 × magnification microscope; (3) selecting 5 fields with the most CD8^+^ T cell infiltration from the 10 fields in the previous step; and (4) taking the average value from these 5 fields as the number of CD8^+^ T cells on the slide. Finally, high and low expression of each molecule was divided by the mean value from the corresponding IHC evaluation results for each patient. To evaluate IHC results of CD38^+^ plasma cells (PCs), a semi-quantitative PC score was employed. In detail, the number of PCs was calculated by combining staining intensity and percent positive area. Staining intensity ranged from 0 to 3 (0, negative; 1, weak; 2, moderate; and 3, strong). The following scores were applied: score = 0, 0–5% of immune cells; score = 1, 6–20% of immune cells (moderate or strong intensity) or 6–100% of immune cells (weak intensity) (B); score = 2, 21–75% of immune cells (moderate or strong intensity); and score = 3, 76–100% of immune cells (moderate or strong intensity) (D) [23]. Low CD38^+^ PC density was defined as a PC score of 0 or 1. High CD38^+^ PC density was defined as a PC score of 2 or 3. Positive or negative expression of CD23 determined if germinal centers (GCs) were present in TLSs.

### Bioinformatics analysis

The expression profiles of RNA-sequencing data for EC were downloaded as Excel files from University of California–Santa Cruz (UCSC) Xena (https://xenabrowser.net/datapages/) in March 2020; they were based on raw data derived from the latest data from the Cancer Genome Atlas (TCGA). The mRNA gene data were transformed using the standard fragments per kilobase of transcript per million mapped reads standard method. The corresponding clinical information and survival data were also downloaded in the same way. High and low expression of each target gene was based on the median value of RNA-seq expression. The relationship between gene expression and PFS or OS was analyzed.

### Statistical analysis

All statistical analyses were performed using SPSS (version 23.0; SPSS Inc., Chicago, IL, USA) and GraphPad Prism (version 7.0; GraphPad Software Inc., San Diego, USA). Student’s *t* test and the Mann–Whitney U test were used to compare continuous variables. Pearson’s chi-squared test and Fisher’s exact test were used to compare categorical variables. Continuous variables with normal distributions were presented as means ± standard deviation (SD). Non-normally distributed variables were presented as medians ± interquartile range (IQR) [24]. Survival analysis was performed with Kaplan–Meier curves, which were compared using the log-rank test. The association between each variable and survival was evaluated in a univariate Cox regression model. All variables with *P* < 0.05 and other meaningful variables based on the univariate analysis were included in a multivariate Cox proportional hazards regression model. Associations were evaluated based on hazard ratios (HRs) and 95% confidence intervals (CIs). Statistical significance was set at *P* < 0.05.

## Results

### Presence of TLS in EC and TLS classification

**Fig. 1:**
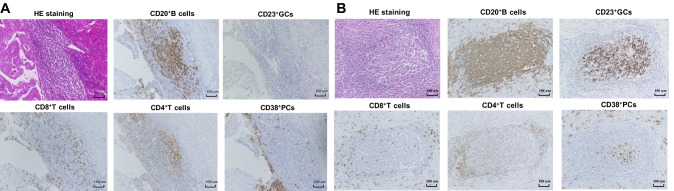
Representative cases of TLS formation and maturation stage. TLS consisted of CD20^+^ B cell-rich area, CD8^+^T cells, CD4^+^T cells, and PCs. TLS can be divided into two stages according to the morphological structure and expression of CD23: **A** early stage, characterized by diffused TLS without CD23^+^GC; **B** mature stage, characterized by aggregated TLS with CD23^+^GC. (Abbreviations: TLS, tertiary lymphoid structures; HE: Hematoxylin–eosin; GC, germinal center; PC: plasma cell)

Figure S1 shows the flow diagram for patient selection. Ultimately, 104 patients with EC who met inclusion criteria were included in this study. We clearly observed some TLSs, as clusters of B cells, both inside tumors and in tumor stroma. Except for areas rich in CD20^+^ B cells, TLSs consisted of CD8^+^ T cells, CD4^+^ T cells, and CD38^+^ PCs, with or without GCs (Fig. 1). B cells were the most dominant TLS component.

**Fig. 2:**
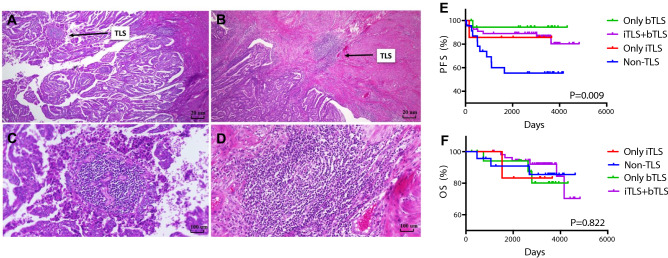
The TLS classification and relationship with survival outcomes characterized by TLS location. TLS were grouped into tumor infiltrative TLS (iTLS) (**A** and **C**) and tumor border TLS (bTLS) (**B** and **D**) according to the location of TLS; The PFS (**E**) and OS (**F**) of overall included patients characterized by TLS location. (Abbreviations: PFS, progression-free survival; OS, overall survival; TLS, tertiary lymphoid structures)

TLS stage and location were evaluated. TLSs can be divided into two stages according to morphological structure and CD23 expression, as shown in Fig. 1: early stage, characterized by diffuse TLSs without CD23^+^ GCs and mature stage, characterized by aggregated TLSs with CD23^+^ GCs. TLS was also grouped into tumor infiltrative TLSs (iTLSs) and tumor border TLS (bTLSs) by location, as shown in Fig. 2. iTLSs were relatively rare, but bTLSs were nearly ubiquitous.

### Comparison of TLSs and TILs in EC, normal endometrium, and AEH

**Fig. 3:**
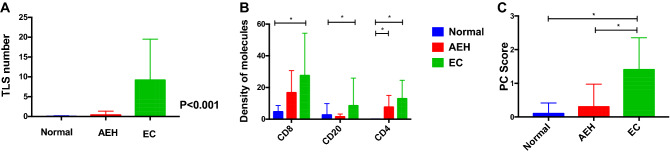
Comparison of TLS and TIL in EC, AEH, and normal endometrium. TLS highly existed in endometrial cancer than normal endometrium (*N* = 10) and AEH (*N* = 10) (**A**); The expression of CD8^+^T cells (B), CD20^+^ B cells (B), CD4^+^ T cells (**B**) and PCs (**C**) in EC patients were significantly higher than normal patients and AEH patients. (Abbreviations: TLS, tertiary lymphoid structures; EC, endometrial cancer; AEH, atypical endometrium hyperplasia; PC, plasma cell)

No TLSs were observed in patients with normal endometrium. TLSs were observed in only two patients with AEH. TLSs were more common in patients with EC than in patients with normal endometrium or AEH (*P* < 0.001) (Fig. 3), which indicated that TLS is a particular structure in tumor tissues. The number of CD8^+^ T cells (*P* < 0.001), CD20^+^ B cells (*P* < 0.001), CD4^+^ T cells (*P* < 0.001), and the density of CD38^+^ PCs (*P* < 0.001) were significantly higher in patients with EC than in patients with normal endometrium or AEH.

### Clinicopathological characteristics and prognostic value of TLS

The 104 patients with EC were divided into two groups (Table 1) based on the analysis of slides: the TLS group (*N* = 81, 77.9%) and the no-TLS group (*N* = 23, 22.1%). No significant differences were observed between groups in age (*P* = 0.469), histology (*P* = 0.611), International Federation of Gynecology and Obstetrics (FIGO) stage (*P* = 0.266), adjuvant chemotherapy (*P* = 0.535), and risk factors except positive ascites fluid cytology (*P* = 0.001). Therefore, almost all the variables were similar in the survival analysis.

**Table 1:** The clinical and pathological characteristics between TLS and non-TLS group in overall included patients

Characteristics	Total (*N* = 104)	Non-TLS group (*N* = 23)	TLS group (*N* = 81)	*P*
*Age (Mean* ± *SD)*		57.3 (± 9.1)	59.2 (± 11.1)	0.469
≤ 50 year	20 (19.2)	15 (65.2)	46 (56.8)	
> 50 year	84 (80.8)	8 (34.8)	35 (43.2)	
*Histology*				0.611
G1	36 (34.6)	11 (47.8)	25 (30.9)	
G2	15 (14.4)	3 (13.0)	12 (14.8)	
G3	27 (26.0)	5 (21.7)	22 (27.2)	
Serous	16 (15.4)	3 (13.0)	13 (16.0)	
Mixed type	10 (9.6)	1 (4.3)	9 (11.1)	
*FIGO stage*				0.266
I	67 (64.4)	14 (60.9)	53 (65.4)	
II	8 (7.7)	1 (4.3)	7 (8.6)	
III	26 (25.0)	6 (26.1)	20 (24.7)	
IV	3 (2.9)	2 (8.7)	1 (1.2)	
*Tumor size*				0.333
≤ 2 cm	21 (20.2)	3 (13.0)	18 (22.2)	
> 2 cm	83 (79.8)	20 (87.0)	63 (77.8)	
*Invasion depth*				0.329
None or < 1/2	59 (56.7)	11 (47.8)	48 (59.3)	
≥ 1/2	45 (43.3)	12 (52.2)	33 (40.7)	
*Positive LVSI*	38 (36.5)	9 (39.1)	29 (35.8)	0.770
*Positive parametrial involvement*	18 (17.3)	4 (17.4)	14 (17.3)	0.990
*Positive lymph node metastasis*	18 (17.3)	3 (13.0)	15 (18.5)	0.757
*Positive distant metastasis*	15 (14.4)	6 (26.1)	9 (11.1)	0.071
*Positive ascites cytology*	20 (19.2)	10 (43.5)	10 (12.3)	0.001*
*Positive MELF pattern*	12 (11.5)	4 (17.4)	8 (9.9)	0.320
*Receiving pelvic/para-aortic lymphadenectomy*	88 (84.6)	20 (90.0)	68 (84.0)	0.724
*Receiving adjuvant treatment*	62 (59.6)	15 (65.2)	47 (58.0)	0.535
*Survival outcome*				
Recurrence	20 (19.2)	10 (43.5)	10 (12.3)	
Death	13 (12.5)	3 (13.0)	10 (12.3)	

Data are presented as number (%) or mean (± SD) or median (± IQR). (Abbreviations: TLS, Tertiary lymphoid structures; LVSI, lymphovascular space invasion; MELF, microcystic, enlarged and fragmented.*p < 0.05)

**Fig. 4:**
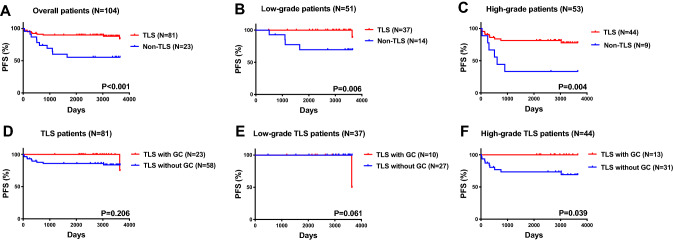
The endometrial cancer patients with TLS were associated with beneficial survival outcomes. **A** The PFS of overall included patients characterized by the presence of TLS; the PFS of overall patients with low-grade histology (**B**) and high-grade histology (**C**) characterized by the presence of TLS; **D** the PFS of 81 TLS patients characterized by the presence of GC; the PFS of TLS patients with low-grade histology (**E**) and high-grade histology (**F**) characterized by the presence of GC. (Abbreviations: PFS, progression-free survival; OS, overall survival; TLS, tertiary lymphoid structures; GC, germinal center)

In univariate analysis, eight factors were associated with PFS, and two factors were associated with OS (Table 2). In multivariate analysis, high-grade histology was associated with inferior PFS (HR, 3.729; 95% CI, 1.212–11.474; *P* = 0.022) and OS (HR, 5.121; 95% CI, 1.092–24.010; *P* = 0.038). TLS absence was independently associated with PFS in patients with EC (HR, 0.154; 95% CI, 0.044–0.536; *P* = 0.003), but not OS. Figure 4 shows the Kaplan–Meier curves for PFS (panel A) for all study patients. We evaluated the relationship between the presence of TLSs and survival outcomes by histology subgroup. In the low-grade histology (*P* = 0.006) (Fig. 4B) and high-grade histology (*P* = 0.004) (Fig. 4C) subgroups, patients with TLSs had better PFS than patients without TLSs. There were positive correlations between intratumoral CD20 + B cells and intratumoral CD8 + T cells, intratumoral CD4 + T cells, as well as plasma cells in patients with low-grade histology (Figure S2).

**Table 2:** The univariate and multivariate analysis of factors associated with PFS and OS in overall included patients

Factors	N	Univariate analysis						Multivariate analysis					
		PFS			OS			PFS			OS		
		HR	95%CI	*P*	HR	95%CI	*P*	HR	95%CI	*P*	HR	95%CI	*P*
*Age*				0.466			0.596						
≤ 50 year	20	1			1								
> 50 year	84	1.579	0.462–5.391		1.503	0.333–6.792							
*Histology*				0.018*			0.037*			0.022*			0.038*
High-grade	51	1			1			1			1		
Low-grade	53	3.396	1.233–9.354		5.034	1.102–2.905		3.729	1.212–11.474		5.121	1.092–24.010	
*FIGO stage*				< 0.001*			0.092			0.230			
Early-stage	75	1			1			1					
Late-stage	29	7.411	2.826–19.433		2.557	0.857–7.635		2.644	0.541–12.914				
*Adjuvant treatment*				0.068			0.817						
No	42	1			1								
Yes	62	2.773	0.926–8.299		0.874	0.279–2.732							
*Tumor size*				0.230			0.937						
≤ 2 cm	21	1			1								
> 2 cm	83	2.447	0.567–10.553		0.949	0.259–3.479							
*Invasion depth*				0.001*			0.752			0.578			
None or < 1/2	59	1			1			1					
≥ 1/2	45	6.216	2.076–18.616		1.193	0.400–3.555		1.359	0.461–4.004				
*LVSI*				0.004*			0.312			0.742			
No	66	1			1			1					
Yes	38	3.896	1.553–9.779		1.760	0.588–5.268		1.218	0.377–3.934				
*Parametrial involvement*				0.316			0.523						
No	86	1			1								
Yes	18	1.678	0.610–4.620		1.524	0.418–5.554							
*LN metastasis*				0.002*			0.965			0.218			
No	86	1			1			1					
Yes	18	4.009	1.633–9.844		0.963	0.213–4.360		3.415	0.594–9.821				
*Distant metastasis*				< 0.001*			0.030*			0.496			0.119
No	89	1			1			1			1		
Yes	15	7.110	2.922–17.298		3.726	1.139–12.191		2.415	0.475–4.649		2.634	0.779–8.908	
*Ascites*				0.005*			0.977			0.960			
No	84	1			1			1					
Yes	20	3.511	1.453–8.483		1.020	0.271–3.836		0.965	0.240–3.885				
*MELF*				0.763			0.410						
No	92	1			1								
Yes	12	1.208	0.354–4.126		0.041	0.000–82.362							
*TLS*				0.002*			0.806			0.003*			0.756
No	23	1						1			1		
Yes	81	0.253	0.105–0.609		0.850	0.233–3.098		0.154	0.044–0.536		0.808	0.210–3.102	

*PFS* Progression-free survival; *OS* overall survival; *LVSI* lymphovascular space invasion; *MELF* microcystic, enlarged and fragmented; *TLS* tertiary lymphoid structures.*p < 0.05

In addition, we compared the relationship between the TLS stage and survival outcomes in 81 TLS patients. There was no significant difference of PFS in overall TLS patients (*P* = 0.206, Fig. 4D) and low-grade histology subgroup (*P* = 0.061, Fig. 4E). However, the TLS patients with GCs had better PFS than those without GCs (*P* = 0.039, Fig. 4F) in high-grade histology. Moreover, by TLS location, there were four subtypes of patients. Figure 2 shows that patients with only iTLSs had the best PFS, followed by patients with both iTLSs and bTLSs. Patients without TLSs had the worst PFS (*P* = 0.009). There were no significant differences in OS by TLS location (*P* = 0.822). Similar results were obtained for PFS.

### *Correlation between CD20*^+^*B cells and TLS, as well as other TILs*

**Fig. 5:**
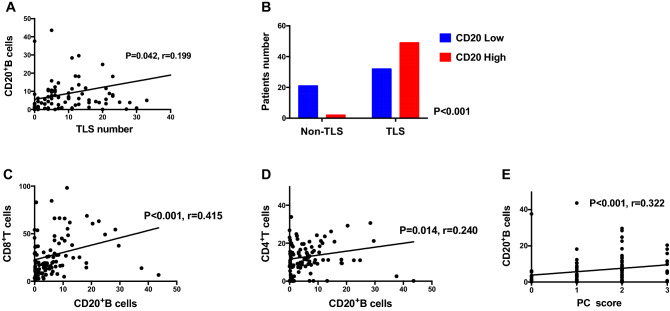
The number of CD20^+^B cells in TLS patients was increased and positively correlated with other TILs. **A** The number of intratumoral CD20^+^B cells was improved along with an increase in TLS number; **B** the number of intratumoral CD20^+^B cells in TLS patients was significantly higher than non-TLS patients; there were positive correlations in each of comparison between CD20^+^B cells and CD8^+^T cells (**C**), CD4^+^T cells (**D**), as well as PCs (**E**). (Abbreviations: TLS, tertiary lymphoid structures; PC, plasma cells)

**Fig. 6:**
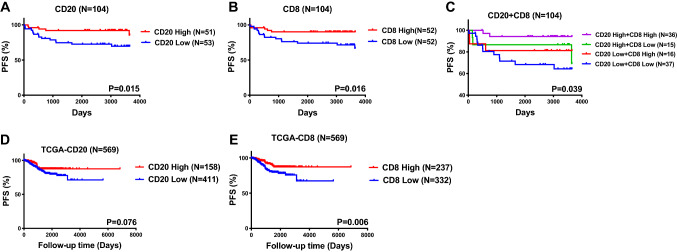
The large number of CD20^+^B cells and CD8^+^T cells was associated with favorable survival outcomes in endometrial cancer patients. The PFS of overall included patients characterized by the number of intratumoral CD8^+^T cells (**A**) and CD20^+^B cells (**B**); the PFS of overall included patients characterized by the combined number of intratumoral CD20^+^B cells and CD8^+^T cells (**C**); the PFS of endometrial cancer patients characterized by the number of CD20^+^B cells (**D**) and CD8^+^T cells (**E**) in TCGA database. (Abbreviations: PFS, progression-free survival; TLS, tertiary lymphoid structures; PC, plasma cells)

CD8^+^ T cells, CD20^+^ B cells, CD4^+^ T cells, and CD38^+^ PCs were common in endometrial tumors. The number of CD20^+^ B cells increased as the number of TLSs increased (Fig. 5A). The TLS group had significantly larger number of CD20^+^B cells than the no-TLS group (Fig. 5B). The number of CD20^+^ B cells was positively correlated with CD8^+^ T cells (*P* < 0.001, *r* = 0.415), CD4^+^ T cells (*P* = 0.014, *r* = 0.0.240), and CD38^+^ PCs (*P* < 0.001, *r* = 0.322) (Fig. 5C, D, and E). Figure 6 and Table S2 show the relationships between intratumoral TILs and survival outcomes in all study patients and by histology subgroup. The larger number of CD20^+^ B cells (*P* = 0.015), CD8^+^ T cells (*P* = 0.016), and higher density of CD38^+^PCs (*P* = 0.012) were associated with better PFS, while the smaller number of CD4^+^ T cells was associated with better OS (*P* = 0.031). Similar results were observed with data from the TCGA database. The larger number of CD20^+^ B cells (*P* = 0.076) and CD8^+^ T cells (*P* = 0.006), respectively, tended to be associated with better PFS (Fig. 6D and E).

## Discussion

Recently, several immune checkpoint inhibitors are the focus of a new treatment strategy for treating different types of gynecologic malignancies [25–27]. TLSs, as clusters of immune cells, play a key role in the TME and is related to beneficial survival outcomes in several solid tumors. TLS upregulation may lead to significant antitumor responses. Cabrita et al. indicated that TLSs play a key role in the immune microenvironment in melanoma, by conferring distinct T cell phenotypes [28]. Figenschau et al. reported that breast carcinomas frequently contain TLSs, and the presence of these structures is associated with aggressive tumors [29]. In HER2-positive and triple-negative breast cancer, the presence of TLSs is associated with superior survival [30]. Caro et al. found that TLSs cooperate with TILs in a coordinated antitumor immune response in patients with low-risk, early-stage colorectal cancer [31]. Dieu-Nosjean et al. first reported the presence of TLSs in non-small cell lung cancer (NSCLC); they named those structures tumor-induced bronchus-associated lymphoid tissue (Ti-BALT). Mature dendritic cell (DC-LAMP^+^) is a specific marker of Ti-BALT. And the number of DC is highly correlated with the infiltration of CD4^+^ and T-bet^+^ Th1 cells in tumors, which is also related to survival outcomes [18]. Since there is a close relationship between TLSs and antitumor response in tumor samples, we naturally focused on TLS in EC. We found that the presence of TLSs in EC is associated with favorable survival outcomes.

Traditional secondary lymphoid organs (SLOs), including the spleen, lymph nodes, and tonsils, are important parts of the immune system [32]. TLSs have a similar structure as typical SLOs, with some slight differences. TLSs consist of B cell-rich regions, as well as T cells, PCs, follicular helper T (Tfh) cells, follicular dendritic cells (FDCs), and GCs [33]. There are also special lymph vessels characterized by high endothelial venules (HEVs) [34]. In chronic inflammatory conditions, such as autoimmune diseases, chronic infections, chronic graft rejection, and tumors, TLSs can serve as effective sites for tumor reactions in the TME and trigger an inflammatory response by infiltrating immune cells independently of SLOs [35].

TLS were divided into different stages according to CD23 expression. TLSs in the mature stage were associated with better survival outcomes. The presence of GCs within TLSs is correlated with exacerbated autoimmune response due to the generation of autoreactive B cells and HEVs. Other researchers have reported similar findings. Florian et al. divided TLSs into three subtypes based on the number of FDCs and mature B cells: (1) early TLSs, composed of diffuse, mixed B cells and T cells or dense lymphocyte clusters without FDCs and GC reactions; (2) primary follicular-like TLSs, which are dense lymphocyte clusters with FDCs but no GC reactions; and (3) secondary follicular-like TLSs, which are dense lymphocyte clusters with FDCs and active GC reactions [21]. Similarly, Silina et al. evaluated 138 patients with NSCLC and divided TLSs into three to four subtypes based on CD21 and CD23 expression [36]. They found that GC formation was impaired and TLS number had no prognostic value in patients treated with neoadjuvant chemotherapy. GCs gradually grow as TLSs develop, eventually becoming activated mature GCs.

Except the stage, TLSs can be classified by tumor location. In our study, patients with iTLSs had better survival outcomes than patients with bTLSs. Hiraoka et al. have also reported that there were two different localizations of pancreatic ductal carcinoma-associated TLSs, intratumoral and peritumoral. iTLS was associated better outcomes, independent of other survival factors. This finding can be explained by the antitumor microenvironment present in tumor tissues with intratumoral TLSs, which was suggested to be in an active state of cellular immune reaction and B cell reaction, as determined by the presence of TILs and tumor cytokines. These subtypes and classifications of TLSs can help us understand the relationships between the role of an immune reaction and antitumor ability in the TME.

Because TLS is an independent factor affecting survival, we further investigated how infiltrating immune cells around TLSs regulate the TME. We found a strong positive correlation between TLSs and intratumoral CD20^+^ B cells. When we evaluated the role of CD20^+^ B cells in survival, strikingly, high number of CD20^+^B cells was associated with favorable PFS. Thus, we hypothesize that B cells lead to a beneficial survival effect associated with TLSs. B cell-related pathways play a key role in the generation and formation of TLSs. At present, the B cell-related pathways associated with TLSs, known as the CCL19/CCL21/CCR7 axis or the CXCL13/CXCR5 axis, are attracting much attention [15]. The chemokine family related to B cells is necessary and sufficient for inducing TLS formation. It has been reported that the CCL19/CCL21/CCR7 axis functions in immune cells and helps cells migrate to SLOs or other tumor sites and activate the host cell response [37]. CCL19 or CCL21 produced by tumor cells is associated with tumor invasion and immune tolerance [38]. The CXCL13/CXCR5 axis is activated by the interaction of B cells and Tfh cells to accelerate the GC reaction; it participates in the migration of tumor B cells and Tfh cells in the TME [39]. In a recent report, TLSs-related gene signature (including CXCL13, CCL19) score was relatively higher in DNA polymerase epsilon (POLE) and microsatellite instability (MSI) subtypes than in other subtypes in TCGA data set of EC [40] And we could verify this result with another data set of endometrial cancer (*n* = 100, Clinical Proteomic Tumor Analysis Consortium [CPTAC]) [41] by the same method as the previous article [40] with single sample gene set enrichment analysis, and we also could get a same tendency of TLSs distribution in POLE and MSI subtypes (data not shown).

DCs, B cells, and Th17 cells are capable of producing these cytokines and are important for the special lymphoid tissue inducer cells during TLS formation [42]. Therefore, B cells and B cell-related pathways need to be further explored as new targets for immunotherapy. How to induce the formation of TLSs and how to inhibit tumorigenesis with TLSs via B cell-related pathways are future research directions.

## Conclusion

In conclusion, TLSs exist in endometrial tumor tissue and are associated with favorable survival outcomes. TLS absence is an independent risk factor for disease progression in patients with EC. TLSs consist of areas rich in CD20^+^ B cells, CD8^+^ T cells, CD4^+^ T cells, and CD38^+^ PCs. TLSs can be divided into two stages according to the number of CD23^+^GCs, and the TLS patients with GCs had better PFS than those without GCs in high-grade histology. Patients with TLSs had significantly higher CD20^+^ B cell number than patients without TLSs. High CD20^+^ B cell number was associated with better PFS. TLSs play an important role in the human immune system in EC. TLSs and corresponding B cell pathways may become new antitumor targets after the T cell therapy revolution.

### Supplementary Information

Below is the link to the electronic supplementary material.

Supplementary file1 (PDF 219 KB)

## Data Availability

All data sources described in this study are directed at the corresponding author.
