# Supplementary material for: Tertiary lymphoid structures are associated with favorable survival outcomes in patients with endometrial cancer
Source: Cancer Immunol Immunother. 2021 Oct 23;71(6):1431–42. doi: 10.1007/s00262-021-03093-1 (PMC9123039; doi:10.1007/s00262-021-03093-1)
Supplement: Supplementary file 1 — Supplementary file1 (PDF 219 KB) [file 262_2021_3093_MOESM1_ESM.pdf]

Supplementary tables

Table S1. The product information and immunohistochemistry conditions of each molecule in this study.

**Antibody**	**CD8**	**CD20**	**CD4**	**CD38**	**CD23**
**Company**	HISTOFINE	NOVUS	Cell Marque	Arigo	Abcam
**Catalog number**	413201	931-2CS161219	0000056827	61420	3275127-4
**Clonality**	Monoclonal	Monoclonal	Monoclonal	Monoclonal	Monoclonal
**Clone**	C8/144B	L26	SP35	SP149	SP23
**Host**	Mouse	Mouse	Rabbit	Rabbit	Rabbit
**Storage**	2-8℃	2-8℃	2-8℃	-20℃	-20℃
**Antigen retrieval condition**	Boiled at 125 °C in a pressure cooker for 10 min	Boiled at 700w in a microwave for 15 min followed by cooling at RT for	Boiled at 125 °C in a pressure cooker for 15 min	Boiled at 700w in microwave for 10 min followed by cooling at RT for 20 min	Boiled at 700w in microwave for 10 min followed by cooling at RT for 20 min
**Primary dilution**	No dilution	1：500	1:100	1:100	1:100
**Dilution solution**	1x PBS	1x PBS	Antibody diluent (Dako, #S3022)	1x PBS	TBS with 1x BSA
**DAB time**	3min 30sec	1min	5min	4min	2min 30sec

書式を変更: 蛍光ペン

削除: **Lot**

Table S2. The relationship between tumor infiltrating lymphocytes and survival outcomes in overall included patients.

Molecular expression	N	PFS			OS		
		HR	95%CI	P	HR	95%CI	P
**Intra CD20⁺B cells**				0.015*			0.158
Lower than 50%	53	1			1		
Higher than 50%	51	0.306	0.127-0.737		0.439	0.148-1.301	
**Intra CD20⁺B cells of low-grade**				0.182			0.143
Lower than 50%	25	1			1		
Higher than 50%	26	0.254	0.044-1.463		0.126	0.008-2.013	
**Intra CD20⁺B cells of high-grade**				0.049*			0.364
Lower than 50%	28	1			1		
Higher than 50%	25	0.337	0.122-0.927		0.571	0.175-1.861	
**Intra CD8⁺T cells**				0.016*			0.162
Lower than 50%	52	1			1		
Higher than 50%	52	0.308	0.128-0.741		0.441	0.149-1.309	
**Intra CD8⁺T cells of low-grade**				0.037*			0.204
Lower than 50%	27	1			1		
Higher than 50%	24	0.154	0.026-0.896		0.460	0.140-1.510	
**Intra CD8⁺T cell of high-grade**				0.063			0.204
Lower than 50%	25	1			1		
Higher than 50%	28	0.377	0.136-1.048		0.460	0.135-1.571	
**Intra CD4⁺T cells**				0.067			0.003*
Lower than 50%	53	1			1		
Higher than 50%	51	2.301	0.953-5.556		7.096	2.371-21.230	
**Intra CD4⁺T cells of low-grade**				0.783			0.478
Lower than 50%	35	1			1		
Higher than 50%	16	0.741	0.098-5.571		2.626	0.118-58.350	
**Intra CD4⁺T cells of high-grade**				0.159			0.031*
Lower than 50%	18	1			1		
Higher than 50%	35	2.407	0.847-6.844		6.868	2.063-22.870	
**CD38⁺PCs**				0.012*			0.190
Lower than 50%	50	1			1		
Higher than 50%	54	0.295	0.122-0.709		0.473	0.160-1.405	
**CD38⁺PCs of low-grade**				0.517			0.144
Lower than 50%	24	1			1		
Higher than 50%	27	0.558	0.096-3.244		0.126	0.008-2.027	
**CD38⁺PCs of high-grade**				0.010*			0.474
Lower than 50%	26	1			1		
Higher than 50%	27	0.222	0.080-0.611		0.651	0.199-2.129	

Combined molecular expression	P value of PFS	P value of OS
**Intra CD20⁺B and CD8⁺T cells**	0.039*	0.412
**Intra CD20⁺B and CD8⁺T cells of low-grade**	0.225	0.419
**Intra CD20⁺B and CD8⁺T cells of high-grade**	0.079	0.535

**Intra CD20$^+$B cells and PC**	0.022*	0.394
**Intra CD20$^+$B cells and PC of low-grade**	0.376	0.151
**Intra CD20$^+$B cells and PC of high-grade**	0.113	0.693
**Intra CD8$^+$/CD4$^+$T cells**	0.020*	0.033*
**Intra CD8$^+$/CD4$^+$T cells of low-grade**	0.076	0.109
**Intra CD8$^+$/CD4$^+$T cells of high-grade**	0.142	0.178

(Abbreviations: Intra: intratumor; PFS, progression-free survival; OS, overall survival. *p<0.05.)

Supplementary figures

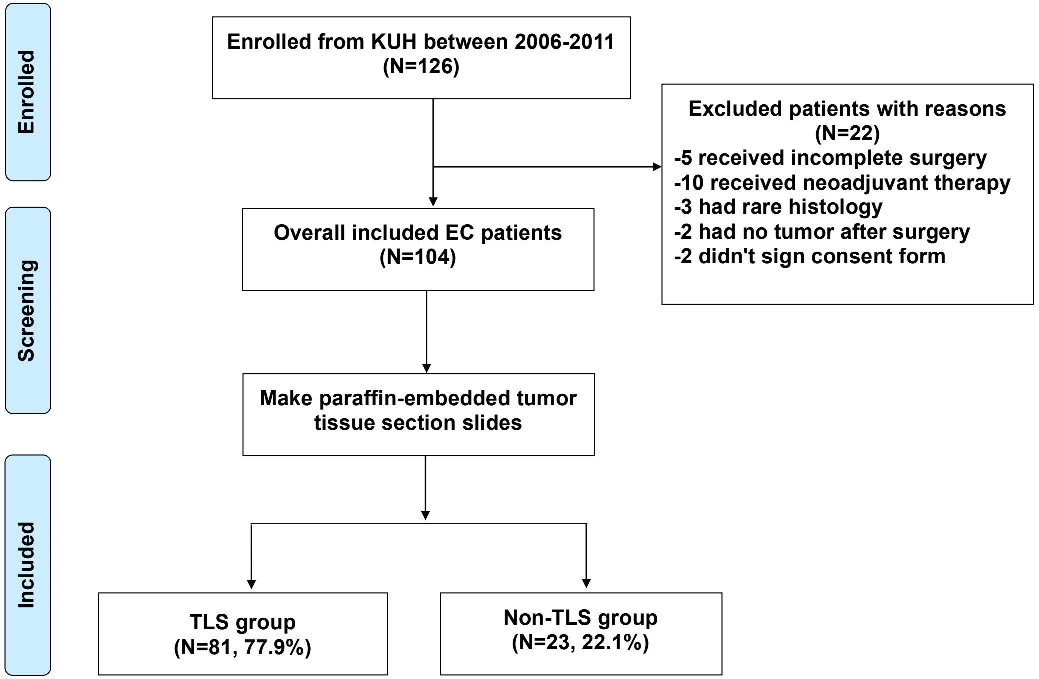

Figure S1. The flow diagram of population collection in this study. (Abbreviations: KUH, Kyoto University Hospital; TLS, tertiary lymphoid structures.)

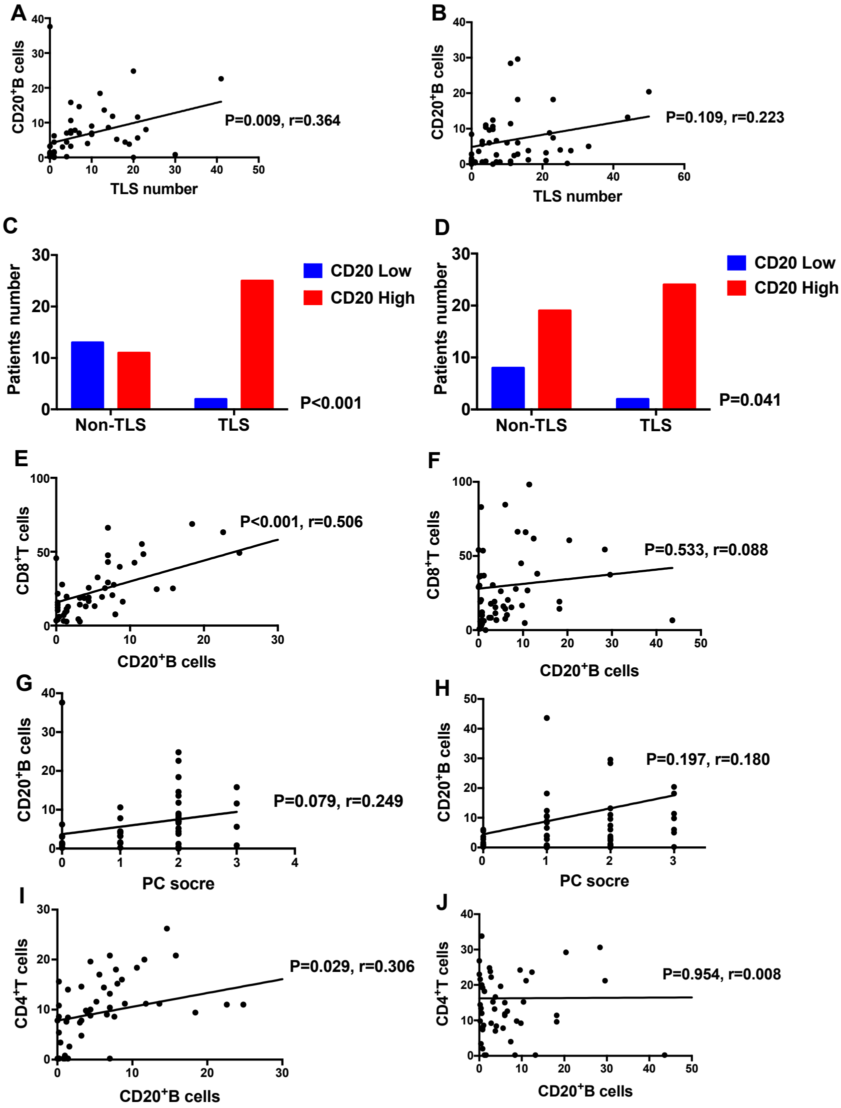

Figure S2. The correlations between CD20⁺B cells and other TILs in patients with low-grade or high-grade histology.

The density of intratumoral CD20+B cells were improved along with an increase in TLS number in patients with low-grade (A) or high-grade histology (B). The expression of intratumoral CD20⁺B cells in TLS patients were significantly higher than Non-TLS patients, respectively in low-grade (C) and high-grade group (D). There were positive correlations between intratumoral CD20⁺B cells and intratumoral CD8⁺T cells (E), intratumoral CD4⁺T cells (G), as well as plasma cells (I) in patients with low-grade histology. There were no correlations between intratumoral CD20⁺B cells and intratumoral CD8⁺T cells (F), intratumoral CD4⁺T cells (H), as well as plasma cells (J) in patients with high-grade histology. (Abbreviations: Intra, Intratumoral; PC, plasma cells.)
